# The Cambridge Centre for Myelin Repair trial Two (CCMR Two): a trial protocol for a phase 2a, randomised, double-blind, placebo-controlled clinical trial of the ability of the combination of metformin and clemastine to promote remyelination in people with relapsing-remitting multiple sclerosis already on disease-modifying therapy

**DOI:** 10.1186/s13063-025-09254-2

**Published:** 2025-12-08

**Authors:** Gioia Riboni-Verri, Christopher E. McMurran, Trisha Mukherjee, Cyrus Daruwalla, Jonathon Holland, Riddhima Gautam, Benson S. Chen, Emma Cutting, Wendi Qian, David MacManus, Declan T. Chard, J. William L. Brown, Alasdair J. Coles, Nick G. Cunniffe

**Affiliations:** 1 Department of Clinical Neurosciences, University of Cambridge, Cambridge, UK; 2 Cambridge Clinical Trials Unit, University of Cambridge, Cambridge, UK; 3 NMR Research Unit, Queen Square Multiple Sclerosis Centre, University College London (UCL) Queen Square Institute of Neurology, London, UK; 4 National Institute for Health Research Biomedical Research Centre, University College London Hospitals NHS Foundation Trust and University College London, London, UK

**Keywords:** Multiple sclerosis, Randomised controlled trial, Remyelination, Visual evoked potential, Magnetisation transfer ratio imaging

## Abstract

**Background:**

In multiple sclerosis (MS), progressive disability occurs following degeneration of demyelinated axons. A tractable approach to delay, prevent or reverse disability progression is through enhancement of endogenous remyelination. Clinical trials have deployed drugs, such as clemastine, to target the rate-limiting step in this process: differentiation of oligodendrocyte progenitor cells (OPCs). Preclinical research has shown that metformin can reverse an age-associated deficit in the responsiveness of OPCs to pro-differentiation factors. The purpose of the Cambridge Centre for Myelin Repair trial Two (CCMR Two) is to evaluate the efficacy of the combination of metformin and clemastine to promote remyelination in people with MS.

**Methods:**

Participants with relapsing-remitting MS (RRMS) will be randomised 1:1 to the combination of metformin and clemastine or matched placebos and followed for 24 weeks of treatment. All participants must be stable on a disease-modifying therapy and have evidence of chronic stable optic neuropathy in at least one eye (defined by P100 latency of the visual evoked potential (VEP) ≥ 118 ms, and the absence of a history of acute optic neuritis in the preceding 2 years). The primary outcome measure will be the change in the P100 latency of the full-field VEP between baseline and week 26. It is planned to recruit a total of 70 participants. This will have 80% power to detect a reduction of 3 ms in VEP P100 latency between the two treatment groups. Secondary outcome measures will examine the change in multifocal VEP and the change in lesional magnetisation transfer ratio (MTR) for lesions stratified by location and tissue-specific cohort baseline lesional MTR values.

**Discussion:**

We set out the trial design, the rationale for participant and outcome measure selection, and the pre-specified analyses. With this trial, we expect to be able to detect the structural and functional consequences of remyelination within a sample size feasible for our single-centre trial.

**Trial registration:**

ClinicalTrials.gov NCT05131828, prior to participant enrolment.

## Administrative information

**Table:** 

Title {1}	The Cambridge Centre for Myelin Repair trial Two (CCMR Two): a trial protocol for a phase 2a, randomised, double-blind, placebo-controlled clinical trial of the ability of the combination of metformin and clemastine to promote remyelination in people with relapsing-remitting multiple sclerosis already on disease-modifying therapy
Trial registration {2a and 2b}	The trial was registered with ClinicalTrials.gov, NCT05131828
Protocol version {3}	Protocol version 5.1. 27 March 2024
Funding {4}	MS Society of the UK
Author details {5a}	Gioia Riboni-Verri^1^ Christopher E McMurran^1^ Trisha Mukherjee^1^ Cyrus Daruwalla^1^ Jonathon Holland^1^ Riddhima Gautam^1^ Benson S Chen^1^ Emma Cutting^1^ Wendi Qian^2^ David MacManus^3^ Declan T Chard^3^ J William L Brown^1,3^ Alasdair J Coles^1^ Nick G Cunniffe^1^ ^1^Department of Clinical Neurosciences, University of Cambridge, Cambridge, United Kingdom ^2^Cambridge Clinical Trials Unit, University of Cambridge, Cambridge, United Kingdom ^3^NMR Research Unit, Queen Square Multiple Sclerosis Centre, University College London (UCL) Queen Square Institute of Neurology, London, United Kingdom and National Institute for Health Research Biomedical Research Centre, University College London Hospitals NHS Foundation Trust and University College London, London, United Kingdom
Name and contact information for the trial sponsor {5b}	Cambridge University Hospitals NHS Foundation Trust and the University of Cambridge Clinical Trial Coordinator: Emma Cutting Email: cuh.neuroscienceCCTU@nhs.net

## Introduction

### Background and rationale {6a}

In multiple sclerosis (MS), which affects > 150,000 people in the UK and is among the commonest causes of disability in young adults, central nervous system inflammation leads to acute axonal injury and demyelination [1–3]. Despite the ability of disease-modifying treatments to effectively reduce this inflammation [4, 5], these immunotherapies have failed to benefit people with non-active (in terms of new inflammation) progressive MS [6], and the greatest unmet clinical need is a neuroprotective treatment with the ability to delay, prevent, or reverse disability progression [7].

The promotion of regeneration of the myelin sheath, through enhancing the process of endogenous remyelination, has emerged as one of the most amenable approaches to meet this challenge [8]. Doing so not only restores nerve conduction, but is also directly protective against degeneration [9–13]. This process occurs as a natural response to demyelination: oligodendrocyte progenitor cells (OPCs) become activated, migrate to areas of damage, proliferate and then differentiate into mature, myelinating oligodendrocytes [14]. Unfortunately, this fails in the majority of people with MS [15], and becomes particularly inefficient with age [16]. A deficit anywhere along this sequence could lead to remyelination failure, but given the majority of MS lesions contain adequate numbers of OPCs and increasing OPC recruitment does not accelerate remyelination in experimental models, it is widely held that a rate-limiting stage is the differentiation of the OPC [17–19].

As a consequence, significant resources have been invested to identify pharmacological targets capable of enhancing this rate-limiting stage [20–22], leading to several clinical trials [23–26]. Clemastine, for instance, after being identified as able to stimulate OPC differentiation and myelination of conical micropillars [21], subsequently demonstrated an improvement in the latency of the full-field visual evoked potential (VEP) in a clinical trial of people with relapsing-remitting MS and chronic stable optic neuropathy [23]. Other noteworthy clinical trials have included the demonstration that an H3-receptor inverse agonist/antagonist yields an improvement in the magnetisation transfer ratio (MTR) characteristics of acute lesions [26], and that a retinoic acid X receptor (RXR) agonist (bexarotene) improves VEP latency and lesional MTR in grey matter regions of the brain [27]. Finally, the monoclonal antibody against Lingo-1 (opicinumab) showed an improvement in VEP latency using a per protocol analysis of participants with acute optic neuritis [24], though no effect was seen in a subsequent phase 2 trial using a composite disability endpoint [25]. At present, the leading outcomes for phase 2 trials are to examine the functional consequences of remyelination using electrophysiology, namely by using visual evoked potentials to interrogate changes in the speed of conduction along the visual pathway [28, 29].

The scientific rationale for this trial rests on the discovery that intrinsic changes take place within OPCs as they age that see them become less responsive to the factors that normally enhance differentiation and remyelination [30]. Ribonucleic acid (RNA) sequencing from young and aged OPCs highlighted a significant contribution from the mammalian target of rapamycin (mTOR) pathway, which can be modulated with the AMP-activated protein kinase (AMPK) agonist metformin. Ensuing experiments demonstrated that in aged rats, 3 months of treatment with metformin caused these animals to remyelinate lesions as well as healthy young animals. This therapeutic effect is mediated through allowing the aged OPCs to become responsive to pro-differentiation factors (for example, agents with an anti-muscarinic action such as clemastine) [30]. This rejuvenating strategy could be particularly important given previous evidence in humans that remyelination might be limited by age [31].

As a result, we set out to design a phase 2a, randomised, double-blind, placebo-controlled clinical trial that would evaluate the ability of the combination of metformin and clemastine to promote remyelination in people with MS. The Cambridge Centre for Myelin Repair trial Two (CCMR Two) tests the ability of this repurposed combination of drugs to reduce the latency of the VEP and to improve the MTR characteristics of chronic lesions stratified by their location and baseline tissue integrity. In this article, we describe our trial design, the rationale for participant and outcome measure selection, and the pre-specified analyses.

### Objectives {7}

The primary objective of CCMR Two is to test the ability of the combination of metformin and clemastine to promote remyelination in demyelinated lesions in people with relapsing-remitting multiple sclerosis (RRMS). Secondary objectives are (i) to assess the efficacy of the combination of metformin and clemastine to promote remyelination as measured by multifocal VEP latency and lesional MTR; and (ii) to survey the tolerability of these drugs in people with RRMS.

### Trial design {8}

CCMR Two is a phase 2a, single-centre, double-blind, randomised, placebo-controlled, parallel-groups add-on trial that compares the combination of metformin and clemastine (maximally tolerated dose of metformin sustained release (SR), up to 1 g twice a day, alongside 5.36 mg clemastine twice daily) against matched placebos for 24 weeks in people with RRMS and chronic stable optic neuropathy. The primary outcome for this superiority trial is the change in full-field VEP latency between baseline and 26 weeks.

## Methods: participants, interventions and outcomes

### Study setting {9}

Participants have a total of seven trial visits to Addenbrooke’s Hospital of Cambridge University Hospitals NHS Foundation Trust in the UK.

### Eligibility criteria {10}

Selection criteria for CCMR Two, alongside the rationale for each point, are shown in Table 1. All participants must be willing and able to give written informed consent and (in the investigator’s opinion) to comply with all trial requirements (including MRI scanning procedures).

**Table 1:** Selection criteria for the CCMR Two trial with corresponding justification for each

**Inclusion criteria**	**Rationale**
Age between 25 and 50 years (inclusive) at time of signing informed consent form*	Upper limit selected to reduce chance that age-related changes at MRI not misidentified as MS lesions. Lower limit to align cohort with the preclinical data showing remyelination failure is age-dependent
Relapsing-remitting multiple sclerosis as per the McDonald 2017 criteria, including an MRI brain satisfying the 2017 radiological criteria [32]	The expectation is that selecting RRMS patients will maximise the number of demyelinated MS lesions with intact axons [8, 17], thereby providing the substrate for remyelination
Full-field visual evoked potential (VEP) P100 latency in at least one eye of ≥ 118 ms	To ensure there is sufficient demyelination in the visual pathway to allow detection of remyelination. 118 ms is 2 standard deviations greater than the mean of our control data, and the same threshold as that previously used in the ReBUILD trial [23]
Kurtzke EDSS step 0.0–6.0*	To maximise the number of intact axons; increased degrees of neurodegeneration would be anticipated at higher levels of baseline disability
At the time of screening, being treated with a stable dose for at least 6 months of a category 1 multiple sclerosis disease-modifying treatment (DMT) or for at least 2 years with a category 2 DMT [5]*†	This allows the regeneration of myelin to be studied in some degree of isolation from active inflammation
**Exclusion criteria**	**Rationale**
Female participants who are pregnant, lactating, or planning pregnancy during the trial Female participants of child-bearing potential whom are unwilling or unable to use one highly effective method of contraception during the trial	Metformin not recommended in pregnancy and is detectable in breast milk. Clemastine should not be given in pregnancy or when breastfeeding
Male participants who are unwilling or unable to use contraception during and for 3 months after the trial	Study reported preconception paternal metformin treatment is associated with major birth defects, particularly genital birth defects in boys [33]
Participants whom have received an investigational drug (including investigational vaccines) or used an invasive investigational medical device within 4 weeks before the screening assessment or is currently enrolled in an interventional investigational trial. Participants who currently use an unlicensed treatment of MS	To maximise chance that any change observed on trial is due to metformin and clemastine, rather than any other investigational product or device
Retinal nerve fibre layer thickness on spectral-domain OCT < 70 μm in the qualifying eye	Below this threshold there are severe decrements in visual function [34]; above this value implies there remain sufficient axons to remyelinate
A clinical episode of optic neuritis in the qualifying eye within the 2 years preceding screening	Following an attack of optic neuritis, VEP latencies are prolonged, but a period of recovery follows for up to 2 years [35, 36]; excluding these make improvements in latency more specific for drug-induced remyelination
Any concomitant use of oxybutynin, monoamine oxidase inhibitors (MAOIs), hypnotics or high-dose opiates	These interact with clemastine. Oxybutynin additionally has potential remyelinating efficacy (though limited blood-brain barrier penetrance)
Significant renal or liver impairment (estimated glomerular filtration rate (eGFR) < 60 mL/min/1.73 m^2^; alanine aminotransferase > 3 times the upper limit of normal)	Metformin very rarely can cause liver dysfunction and the incidence of lactic acidosis rises with impaired renal function
People taking medication for diabetes mellitus	Metformin would alter glycaemic control
People with a diagnosis of epilepsy	Clemastine contraindicated in epilepsy. VEP testing contraindicated in photosensitive epilepsy
Concurrent use of 4-aminopyridine or fampridine	These can improve VEP and saccadic latency [37, 38]
History of prostatic hypertrophy, significant cardiac conduction block, decompensated heart failure, stenosing peptic ulcer, or pyloroduodenal ulceration	Clemastine contraindicated in these conditions
People with a history of alcohol or other recreational drug misuse within the 6 months preceding screening. People unable to avoid alcoholic drinks over the course of the trial	Possibility of increased risk of lactic acidosis with metformin and alcohol
People with rare hereditary problems of galactose intolerance, total lactase deficiency, glucose-galactose malabsorption or people with acute porphyria	As per the summary of product characteristics for metformin
Vitamin B12 deficiency defined as B12 levels of < 150 pg/ml measured at screening	MHRA safety update during the trial highlighted metformin can cause vitamin B12 deficiency
History of major ophthalmologic disease including glaucoma, macular degeneration, and severe myopia (> − 7 Diopters)	Severe ophthalmic disease due to causes outside of demyelinating optic neuropathy might impact on VEP and OCT assessments

*MS *multiple sclerosis, *VEP *visual evoked potential, *SD *standard deviation, *BBB *blood-brain barrier, *EDSS *expanded disability status scale, *OCT *optical coherence tomography, *eGFR *estimated glomerular filtration rate, *DMT *disease-modifying treatment, *MHRA *Medicines and Healthcare products Regulatory Agency

*Age, disability, and disease-modifying treatment category are additionally included alongside biological sex as stratification factors in the randomisation (main text) to ensure matching baseline characteristics between active and placebo groups. †For the purposes of this trial, higher efficacy (category 2) DMTs include ocrelizumab, ofatumumab, natalizumab and alemtuzumab

### Who will take informed consent? {26a}

Written informed consent will be taken prior to any trial-specific procedures, obtained by the investigator or designee. The informed consent form (ICF) is signed by both the investigator and the participant.

### Additional consent provisions for collection and use of participant data and biological specimens {26b}

Participants will agree to their records being inspected by the trial team, regulatory authorities and representatives of the sponsors. They will also agree that their GP be informed of their participation in the trial and be informed of any abnormal investigations during the trial. Optional consent clauses will include the following: (i) the donation of blood samples for research (including storage and future ethically approved research); (ii) permission for DNA from blood samples to be stored and used for future ethically approved research; (iii) the donation of stool samples for research purposes with subsequent storage and use in future ethically approved research; (iv) authorisation for the research team to access relevant medical records for future ethically approved research; (v) agreement for de-identified research data (including copies of MRI scans) and biological samples to be shared with collaborators within and outside the UK and EEA for ethically approved research; (vi) permission to receive the trial newsletter at the end of the trial; and (vii) agreement to allow the trial investigators to contact the participant in the future with respect to further follow-up studies for this trial or related ethically approved studies.

## Interventions

### Explanation for the choice of comparators {6b}

Placebo tablets to match both metformin and clemastine tablets will be supplied by the Sponsor-appointed third party. These will be packaged, labelled, supplied, stored and administered as per their respective active treatment.

### Intervention description {11a}

#### Metformin

Metformin is a biguanide licensed for human use in the management of type 2 diabetes [39]. The primary target of metformin is the respiratory chain complex I [40]. Towards this, the drug induces mild and transient inhibition, increasing the cellular adenosine monophosphate (AMP) to adenosine triphosphate (ATP) ratio, leading to activation of AMPK, which regulates various target genes. It also functions to inhibit the mTOR pathway through both AMPK-mediated and AMPK-independent mechanisms. Ultimately, this leads to reduced hepatic glucose production (inhibits gluconeogenesis and glycogenolysis), increased insulin sensitivity in muscle (improves uptake and use of glucose), delayed intestinal glucose absorption, and reduced total cholesterol, low-density lipoprotein (LDL), and triglyceride levels [41].

Metformin has previously been demonstrated to reduce inflammation in progressive and relapsing experimental autoimmune encephalomyelitis [42], and has been used in an open label trial of 20 people with MS and demonstrated a reduction in the number of new or enlarging T2 lesions compared with placebo [43]. The demonstration that it can also reverse an age-associated barrier to the ability of oligodendrocyte progenitor cells to respond to pro-differentiation factors and enhance remyelination [30] provides a compelling rationale for its use in this trial.

Metformin has been used in clinical practice for over 60 years, and its safety profile is well established. Metformin is contraindicated in those with an estimated glomerular filtration rate (eGFR) < 30 mL/min/1.73m^2^ and the maximum dose is reduced in those with an eGFR between 30 and 60 mL/min/1.73m^2^. As a result, the selection criteria applied to the trial will ensure only those with an eGFR ≥ 60 mL/min/1.73m^2^ can be included. Helpfully, while metformin is an anti-diabetic agent, it never causes hypoglycaemia and requires no monitoring of blood glucose levels. Meanwhile, metformin is generally well tolerated. Gastrointestinal (GI) upset occurs in up to 20%, leading to discontinuation in 5%; however, this is often short lasting and can be minimised by gradual up-titration of the dose [44]. Prolonged-release forms, offering a slower absorption rate, additionally improve tolerability [45–47].

The oft-feared consequence of lactic acidosis with metformin use is usually predictable (in those with renal failure) and is a very rare event—between 3 and 9 events per 100,000 patient years only [48]. However, the summary of product characteristics for metformin highlights that it should be temporarily discontinued in circumstances that may precipitate renal injury (such as severe dehydration and vomiting) or with use of intravenous iodinated contrast.

#### Clemastine

Clemastine is a first-generation (inverse agonist of histamine) anti-histamine that inhibits receptors of the H1 type. It has several licensed indications including allergic rhinitis, hay fever, allergic dermatoses, and urticaria.

After preclinical work demonstrating the potential remyelinating effect of clemastine [21], it was tested in a phase 2 clinical trial [23]. The ReBUILD trial was a single-centre, double-blind, randomised, placebo-controlled, phase 2, crossover trial, which specifically investigated the remyelinating potential of clemastine in patients with RRMS and evidence of chronic demyelinating optic neuropathy. Their inclusion criteria ensured that there was detectable demyelination in the optic pathway (evidenced by a VEP P100 latency ≥ 118 ms in at least one eye), but also sufficient axons to regenerate (with a retinal nerve fibre layer (RNFL) thickness > 70 μm in the qualifying eye when measured with OCT). Meanwhile, the remyelination that might be expected in the natural history of optic neuritis was excluded by selecting only those without a history of acute optic neuritis in the qualifying eye within the last 5 years, or in either eye in the last 6 months. The trial design saw participants divided into two groups, but ensured that all had access to the trial drug. Twenty-five were given 5.36 mg of clemastine twice daily for 90 days followed by placebo for 60 days (group 1), while a further 25 patients were given placebo for 90 days followed by clemastine for 60 days (group 2). The results of this were promising. VEP P100 full-field latency was reduced by 1.7 ms/eye (*p* = 0.0048) in the crossover model. Furthermore, the clinical effect of clemastine was sustained in group 1 after switching to placebo. Thus, the crossover model underestimates the actual effect, later demonstrated to be a 3.2 ms reduction in P100 latency. It also implied a durable change in myelination in the optical pathway. Furthermore, there was a significant improvement in a functional outcome, low-contrast letter acuity, when the delayed treatment analysis was employed.

Clemastine is a safe medication with no serious adverse effects; in the ReBUILD trial, the drug was well tolerated, though it was associated with fatigue. However, higher doses would be limited by its tendency to sedation, which is likely to be problematic in this cohort in whom fatigue is common. The summary of product characteristics for clemastine suggests caution in those with narrow angle glaucoma, prostatic hypertrophy with urinary retention, a history of pyloroduodenal ulceration and cardiac conduction block; participants with these comorbidities will be excluded in CCMR Two.

#### Dosing regimen

Following randomisation, at the baseline visit, the trial drugs will be dispensed from the pharmacy. This will include tablets of clemastine 1.34 mg (or matched placebo) and metformin SR 500 mg (or matched placebo). Participants will be instructed to commence 5.36 mg twice daily of clemastine on day 1 of the trial and continue this for the 24-week treatment period. This dose has been selected as it remains below the licenced maximum dose (8.04 mg twice daily); the same dose has previously been deployed in the ReBUILD clinical trial (with no treatment discontinuation in a cohort of 50 patients with RRMS), and the trial also reports only partial saturation of the target muscarinic receptor (and so we have not trialled a lower dose) [23]. Participants will similarly be instructed to commence 500 mg twice daily of metformin SR on day 1. This will be up-titrated to 500 mg in the morning and 1 g in the evening after a period of 2 weeks. There will be a final dose increase to 1 g twice daily after a period of 4 weeks if tolerated. This dose has been selected as, in the preclinical experiments, the rats consumed 250 mg/kg/day; this translates to approximately 2.5 g per day assuming a body surface area of 1.7 m^2^ [49]. With 2 g/day being the maximum dose recommended of a sustained release preparation of metformin, we have elected not to increase this any further.

### Criteria for discontinuing or modifying allocated interventions {11b}

If adverse events (AEs) occur that are attributable to metformin—most likely gastrointestinal side effects—the trial team may reduce or stop the metformin dose. If symptoms settle, then the dose may be increased again, at the discretion of the trial doctor. In the event of an unrelated illness, such as diarrhoea and vomiting requiring hospitalisation or a requirement for surgery, then both IMPs should be temporarily discontinued. Additionally, the development of deranged liver function (ALT greater than 3 times the upper limit of normal) or renal function (eGFR < 60 mL/min/1.73 m^2^) during the course of the trial would prompt a repeat blood test and suspension of all IMPs for at least 1 week. No dose modifications are permitted for clemastine.

### Strategies to improve adherence to interventions {11c}

Participants will be issued with a dosing diary at their baseline visit to record the number of tablets taken and to indicate any missed doses (and reasons for non-adherence). However, participants will also be asked to return any unused trial drug and a pill count conducted. A cumulative total of 28 missed doses (i.e. 14 days of incomplete doses) over the entire treatment period will trigger a review by the Chief Investigator (CI) for consideration of participant withdrawal.

### Relevant concomitant care permitted or prohibited during the trial {11d}

A requirement for trial eligibility is treatment with a MS DMT. It is expected that participants continue to receive this medication, provided and prescribed by their original physician and neurology service. However, this is not a condition of continued participation. If participants experience AEs or a lack of efficacy of their treatment and wish (or are advised) to terminate or change treatment, they are free to do so.

Throughout the trial, investigators may prescribe any concomitant medications or treatments deemed necessary to provide adequate supportive care, save for those specified in the exclusions for trial participation. Any medication, other than the trial medication taken during the trial, will be recorded in the case report form (CRF).

Prohibited concomitant medications during the trial are hypoglycaemic agents, oxybutynin, monoamine oxidase inhibitors, hypnotics, high-dose opiates, fampridine and 4-aminopyridine. We will advise participants to avoid alcoholic drinks for the duration of the trial.

### Provisions for post-trial care {30}

Participation in the trial ends following completion of the week 28 visit (or earlier in the case of those withdrawing from the trial). Participants will return to routine NHS care, and any unresolved serious adverse events (SAEs) will be followed up clinically until resolution outside of this trial. The University of Cambridge has arranged insurance for negligent harm caused as a result of protocol design and for non-negligent harm arising through participation in the clinical trial. Participants will be reimbursed for travel and parking costs incurred by completing claim forms (with receipts) at each trial visit. There is no provision for participants to continue on metformin and clemastine—or to start on active treatment—following the end of this trial.

## Outcomes {12}

### Primary outcome: full-field VEP

The primary outcome measure of CCMR Two will be the change in the P100 latency of the full-field VEP between baseline and week 26 for each eye with a baseline latency of ≥ 118 ms. The latter timepoint, 2 weeks removed from the end of treatment (week 24), has been selected to ensure that more than five half-lives of the IMP have passed prior to the final VEP assessment; in this way any change in latency cannot be attributed to a direct effect of the IMP on conduction velocity (making any changes more specific to variation in myelination).

### Secondary outcomes: multifocal VEP

A secondary outcome of the trial is to interrogate the change in multifocal VEP latency, between baseline and week 26, for those eyes with delayed latency (≥ 151 ms) at baseline. While full-field VEP is a sensitive tool for detecting latency abnormalities in the visual pathway [50] and has been deployed in several remyelination trials [23, 24], limitations of full-field VEP include macular overrepresentation in the visual cortex and the propensity for conventional electrode placement (frontal-occipital) to favour the response from the lower visual field; small and localised areas of demyelination are often not detected [51–53]. Additionally, as the upper visual field projects to the lower bank (lingual gyrus) of the sulcus calcarinus, while the lower visual field projects to the upper bank (cuneus gyrus), the full-field VEP is susceptible to the phenomenon of phase cancellation: the cortical dipoles from the upper and lower hemifields are almost opposite, resulting in a cancellation effect on amplitude in an unaffected eye [54]. Consequently, a corollary of demyelination in the visual pathway can be that the recorded signal appears larger due to less cancellation effect. Multifocal VEP (MF-VEP) mitigates against these problems by simultaneously stimulating multiple individual regions of the visual field and extracting the unique signals corresponding to each. This allows for a potentially more precise analysis of latency and amplitude abnormalities in people with optic neuropathy [55–57]. MF-VEP has previously been deployed in a substudy of the RENEW trial [58] and is now being used in remyelination trials as an outcome measure, such as in the clinical trial of nanocrystalline gold (NCT03536559).

### Secondary outcomes: magnetisation transfer ratio

The co-secondary outcome is the change in lesional MTR, between baseline and week 26, for MS lesions stratified by location and the tissue-specific cohort baseline lesional MTR values. MTR correlates with histopathological demonstration of myelin in MS lesions [59] and our analyses have been selected to account for evidence that the degree of remyelination in an MS lesion is dependent both on its location, potentially being greatest in the grey matter [60–63], and the degree of its demyelination at baseline [27]. We have prioritised chronic lesions in our analysis as remyelination trials centred on acute lesions require frequent MRI scans and large numbers of participants [26].

### Secondary outcomes: safety and tolerability

Each trial visit after the point of informed consent will capture data on adverse events (AEs), which will be evaluated by the investigator to establish seriousness and any relationship with the IMP (causality). AEs and withdrawals will be reported.

### Exploratory outcomes: visual pathway

The trial will evaluate the effects of metformin and clemastine on other measures of neuroprotection and visual function measured both before and after the treatment period: (i) the change in the amplitude of the full-field VEP, between baseline and week 26; (ii) the change in the amplitude of the multifocal VEP, between baseline and week 26; (iii) the change in visual acuity (change in numbers of letters read on the Sloan 100%, 2.5% and 1.25% acuity charts (and corresponding LogMAR score)), between baseline and week 28; (iv) the change in colour vision (change in protan, deutan, and tritan confusion lines at the Cambridge Colour Test), between baseline and week 28; (v) the change in visual fields (change in mean deviation and pattern deviation), between baseline and week 28; and (vi) the change in RNFL and ganglion cell inner plexiform layer thicknesses, between screening and week 28 [64]. In subsidiary research, to be analysed outside of the final study report, we will test the change in saccadic latency parameters [65], between screening and week 28 and the change in latencies of the N75 and N145 latencies of the full-field VEP, between baseline and week 26.

### Exploratory outcomes: MRI

We will report the change in T2 lesion load between baseline and week 26 as a measure of inflammatory activity (albeit potentially clinically silent). Using MTR, the effects of metformin and clemastine will be compared at a voxel level, lesion subcomponent level, and at the whole lesion level (including after stratifying lesions by type). These analyses will be modelled on our previous research which showed remyelination in response to bexarotene varied within and between lesions [63]. A final exploratory outcome will report the change in different MRI biomarkers of remyelination (described below).

### Exploratory outcomes: clinical measures and blood biomarkers

CCMR Two will assess the change in disability (measured by the EDSS step) between week 26 and the screening visit. Blood will be stored (optional) for future research on blood-based biomarkers of remyelination. Change in serum neurofilament light chain, between baseline and week 26, has been included for reporting in the statistical analysis plan.

### Definition of eligible eyes for primary, secondary and exploratory outcome measures

As a point of clarification, as per the selection criteria, eligible eyes will have a RNFL on spectral-domain OCT ≥ 70 μm. In the circumstance of a participant having delayed VEP latencies in both eyes, but RNFL thickness < 70 μm in one of those eyes, only the qualifying (≥ 70 μm) eye would be included in the analysis. Additionally, to be included in the multifocal VEP outcome measure analysis, an eye must additionally have 28 or more segments with recordable signal (at least half of the 56 recordable segments).

### Participant timeline {13}

The screening visit will be divided into two sets of assessments. On the first, after signing of the informed consent form (ICF), the trial team will review the participant’s medical history, imaging and records to confirm the diagnosis of RRMS [32] and their eligibility for inclusion (Table 1). Participants will then have their screening VEP assessments to ensure there is detectable demyelination in the visual pathway. Our data suggest that in the region of 25–50% of potential participants will not pass screening at this point; in the ReBUILD trial, it is noteworthy that 75 failed screening on this same criterion to yield 50 eligible participants [23]. If these assessments are satisfied, the individual would then be invited to return for a second series of screening investigations including blood tests, EDSS assessment, and OCT to ensure the criteria outlined in Table 1 are similarly met; a much lower attrition rate is anticipated at this point.

Following documented permission from the potential participant when signing the ICF, their usual treating neurologist and/or general practitioner (GP) will be contacted to detail their involvement in the trial; if they are not able to be randomised after attending screening, reasons for this will be communicated to both the participant and referring clinician. We will also maintain a log of all who are ineligible for the trial and all eligible participants that will not be randomised because they declined participation. This process will enable generalisation of the trial results in accordance with the Consolidated Standards of Reporting Trials (CONSORT) guidelines of 2010 [66].

Baseline assessments will take place after randomisation and up to 2 weeks prior to commencement of IMP. After the baseline visit, the MRI will be reviewed for quality control. If this passes, the participant will start IMP within 2 weeks as outlined. In the unlikely event of the MRI not passing quality control, it is possible that we would require a repeat MRI scan. This would be scheduled at least 2 days prior to the commencement of IMP.

Participants receive 24 weeks of treatment. Participants are to be seen at Addenbrooke’s hospital after 4 weeks, 12 weeks, 26 weeks, and a final visit at 28 weeks from treatment initiation. A visit window for week 4 and week 12 visits of ± 7 days will be permitted where scheduling participant visits is difficult or in the event of ill health or transport issues. A visit window of + 7 days will be permitted for the week 26 visit if a similar problem arises. A visit window of ± 14 days will be permitted for the week 28 visit.

A summary of participant assessments is presented in Table 2.

**Table 2:** Schedule of assessments for CCMR Two

**Assessment**	**Screening 1**	**Screening 2**	**Baseline**	**Treatment phase**						Week 26	Week 28
**Clinic visit number**	**1**	**2**	**3**	No visit	No visit	**4**	**5**	No visit	No visit	**6**	**7**
**Visit window**	**Day − 70 to − 14**	**Day − 70 to − 14**	**Day − 14 to − 7**	Day 1	Day 15	**Week 4 (± 7 days)**	**Week 12** **(± 7 days)**	Week 24	Week 24	**Week 26 (+ 7 days)**	**Week 28 (± 14 days)**
Informed consent	X			**Start IMP**				**Stop IMP**			
Eligibility assessments	X	X									
Medical and MS history	X										
Physical examination^1^		X								X	
EDSS assessment		X								X	
Concomitant medication check	X		X			X	X			X	
Randomisation		X^2^									
Adverse event assessments		X	X			X	X			X	X
IMP dispensing			X			X	X				
IMP dose increase					X	X					
IMP compliance check						X	X			X	
Safety bloods		X				X	X			X	
Optional research bloods (for storing serum, plasma and whole blood)		X					X			X	
Optional stool sample			X			X			X		
12 lead ECG		X									
Pregnancy test (women of childbearing potential only)		X	X			X	X			X	
Full-field VEP	X		X				X			X	
Multifocal VEP	X		X				X			X	
OCT		X									X
Visual fields			X								X
Visual acuity			X				X				X
Eye tracking		X									X
MRI imaging			X							X^3^	

Trial specific activity is shown for each visit; see text for additional information. ^1^Physical examination to include: height (screening only), weight, pulse, and blood pressure. ^2^Randomisation only to be completed once all screening assessments are complete and eligibility confirmed. ^3^The second MRI can be undertaken at either week 26 (or week 28 visit, if necessary). *MS*, multiple sclerosis; *EDSS*, expanded disability status scale; *IMP*, investigational medicinal product; *ECG*, electrocardiogram; *VEP*, visual evoked potential; *OCT*, optical coherence tomography; *MRI*, magnetic resonance imaging

### Sample size {14}

The primary endpoint is the change in the P100 latency of the VEP between baseline and week 26 for each eligible eye. An average reduction of 3.0 ms/eye in VEP at week 26 between metformin + clemastine (research arm) and placebo (control arm) will be an important treatment effect to detect. There is no evidence for an interaction of VEP between eyes. With a 5% significance level (two-sided), 80% power, and a common standard deviation of 5 ms, 45 eyes in each arm are required to detect a difference of 3.0 ms/eye in the mean change of VEP at week 26 from baseline between the two treatment groups (nQuery). The common standard deviation of 5 ms was based on the ReBUILD study [23], which was approximately 4.1 ms, and added around 20% (4.1 × 1.20 = 4.92) more variation. Allowing for 10% non-compliance, that is 50 eyes per arm: it was originally planned to recruit 50 participants. Following the planned interim analysis meeting held on 28/06/23, the trial sample size increased to 70 participants as around 50% of participants had only one eligible eye at screening. The Independent Data Monitoring Committee (IDMC) supported the plan to increase recruitment to reach the required eligible eyes.

### Recruitment {15}

Potential participants will be identified through specialist neurology clinics at the trial centre or referred to the trial team from other MS/neuroscience centres and hospitals. Candidate participants will then be seen in clinic or contacted via telephone/video call to discuss the trial directly with a member of the trial team and ‘prescreen’ to ensure that the interested participant is likely to fulfil the general criteria to enter the trial and be able to comply with the trial assessments and interventions. The potential participant will be given a copy of the participant information sheet (PIS) and ICF and asked to contact the trial team at an interval of no less than 24 h to confirm if they would like to proceed to screening.

## Assignment of interventions: allocation

### Sequence generation {16a}

Eligible participants will be randomly assigned to metformin + clemastine (active arm) or placebo (control arm) in a 1:1 ratio using a minimisation with random element method. The stratification factors used in the randomisation will be: age (≤ 40 vs > 40 years), baseline MS DMT treatment category (1 vs 2) [5], sex (male vs female), and disability (an EDSS step ≤ 4.0 vs > 4.0).

### Concealment mechanism {16b}

A web-based central randomisation system (Sealed Envelope) will allocate the participant a treatment code that will relate uniquely to a supply of IMP.

### Implementation {16c}

Access to the web-based randomisation system will be via individual user accounts provided to the CI and suitably trained and delegated members of the research team. Immediate allocation of treatment will be performed by the Sealed Envelope system following enrolment by the site investigators, with documentation of the decision sent in a confirmatory email.

## Assignment of interventions: blinding

### Who will be blinded {17a}

Throughout the trial, investigators and participants will be blinded to the treatment allocation. The blind will be protected by means of: identical appearance of tablets for active and placebo drugs, equal numbers of tablets prescribed to participants in each arm, coded drug supplies being provided to the clinical trials pharmacy, and the randomisation list being held securely from the investigator’s team (with provision for unblinding in emergencies if required). Staff in the pharmacy and the trial coordinator will be unblinded to trial treatment and allocation. An additional layer of blinding is added by the anonymisation of VEPs and MRIs, such that they are analysed without reference to any participant information.

### Procedure for unblinding if needed {17b}

The online randomisation system will be used for emergency unblinding. To maintain the overall quality and legitimacy of the clinical trial, treatment code breaks may occur only in exceptional circumstances when knowledge of the actual treatment is essential for further management of the participant. If the investigator or treating healthcare professional believes unblinding is necessary, the investigator will be able to break the treatment code using the randomisation system. Appropriately trained and delegated site staff will be given the necessary access rights and permissions to access this facility. The name, contact details of the unblinder, and reason for unblinding will be recorded within the randomisation system.

## Data collection and management

### Plans for assessment and collection of outcomes {18a}

#### Visual evoked potentials

VEPs will be assessed by means of a Vision-Search Plus system (VisionSearch, Sydney, NSW, Australia). The stimulus protocol for full-field VEP (primary outcome) has been selected to comply with guidelines from the International Society for Clinical Electrophysiology of Vision [67]. The visual stimulus will be generated on a high-resolution liquid crystal display, with a 2 Hz reversing check pattern of size of 60 min of arc. The participant will be seated 50 cm from the screen. All will be optimally refracted for near vision, and pupil dilatation will not be required. Electrical signals will be recorded from a channel formed between gold-cup electrodes (Grass Technologies, West Warwick, RI, USA) positioned frontally in the midline and 2.5 cm above the inion (Fz-Oz); a ground electrode will be placed on the ear lobe, and conductive gel (0.5 ml) injected under each. Between 3 and 5 averaged recordings will be taken per eye, and the weighted average of these used to measure the N75, P100 and N145 latencies and the amplitude between the N75 and P100. Two blinded raters will be responsible for ensuring quality control of each VEP record and for latency and amplitude determination.

For the multifocal VEP (secondary electrophysiology outcome), we will use the aforementioned equipment, this time to monocularly present a stimulus consisting of 56 cortically scaled segments, each containing a 4 × 4 checkerboard reversing in a pseudorandom sequence. Four gold-cup electrodes (Grass Technologies) will be used for bipolar recording: a vertical channel (using electrodes placed in the midline 2.5 cm above and 4.5 cm below the inion) and a horizontal channel (via electrodes placed 4 cm either side of the inion). The resultant VEP signals from each of the 56 segments are amplified 100,000 times and band-pass filtered (1–20 Hz). Amplitude is defined as the largest peak-trough signal from either the vertical or horizontal channel, within an interval of 70–200 ms, while latency is taken as the second peak of this maximum amplitude wave. Segments with no detectable amplitude are assigned an amplitude of 0 nV but are not assigned a latency. The software then averages the amplitudes from each segment and averages the latencies from only those segments with recordable signal. In order to be included in the analysis, the baseline recording must have recordable signal in at least 28 of the 56 segments, and a prolonged baseline latency is defined as being ≥ 151 ms. Progression analysis will then be performed across each of the 56 segments from each of the trial visits by two blinded raters, to determine the change in multifocal-VEP latency and amplitude for each affected eye.

#### MRI

MRI scans will be performed at baseline and at 26 weeks, using a Siemens 3 T Prismafit scanner (Siemens, Erlangen, Germany) with 20-channel head-neck coils. Each scan will include three-dimensional (3D) magnetisation transfer imaging (for the calculation of MTR maps), 3D T1-weighted imaging (for volumetric measures and tissue segmentation), interleaved 2D proton density-weighted and T2-weighted scans (for lesion identification), 3D fluid-attenuated inversion recovery (for lesion identification and contouring of T2 hyperintense lesions) and 3D macromolecular tissue volume imaging (MTV). Sequences sensitive to grey matter lesions will also be collected (double inversion recovery (DIR)) [68, 69]. The 3D images will be registered to halfway space. A baseline lesion mask will be generated automatically from the fluid-attenuated inversion recovery, edited, then propagated to the follow-up and updated with any new or enlarging lesions. New and enlarging lesion identification, contouring of grey matter lesions, and checking will be done by three different masked observers.

#### Optical coherence tomography

Heidelberg Spectralis spectral domain OCT (Heidelberg Engineering, Heidelberg, Germany) with eye tracking function (TruTrack, Heidelberg Engineering) will be used to obtain quantitative retinal thickness data and morphological cross-sectional information. The same Spectralis machine will be used for participant scanning throughout the trial, with scans obtained by experienced OCT operators. Scans of the peripapillary RNFL will be obtained by recording a high-resolution ring scan (diameter 12°) around the disc (ART 100). Macular volumetric scans will be obtained using the standard posterior pole protocol of the Spectralis OCT, comprising 61 single lines of 15 frames (30° × 25° volume scan centred on the fovea) obtained in high-speed mode with ART 16.

The quality of ring and volume scans will be assessed using OSCAR-IB consensus criteria for retinal OCT quality assessment [70]. Two independent and blinded raters will determine macular and RNFL thicknesses on coded scans for each participant.

#### Visual fields

A Humphrey Field Analyser (Carl Zeiss Meditec, Jena, Germany) will be used to conduct visual field testing of each eye at baseline and week 28, deploying the Swedish interactive threshold algorithm (SITA) standard strategy (30-2 protocol). Reliability criteria will be fixation losses of 20% or less, false-positive results of 15% or less, and false-negative results of 33% or less.

#### Visual acuity

Examination of high and low-contrast letter acuity will be performed for each eye separately at baseline, week 12, and week 28, with refractive correction for distance. High contrast letter acuity testing will be completed using Early Treatment Diabetic Retinopathy Study (ETDRS) visual acuity charts on a large ETDRS illuminator cabinet (Precision Vision, Woodstock, IL, USA). Low-contrast letter acuity testing will be completed using retroilluminated 2.5% and 1.25% Sloan Revised low contrast charts (Precision Vision, Woodstock, IL, USA). Each testing round will begin at 4 m. For each test (ETDRS acuity, low-contrast letter acuity 2.5% and 1.25%), the total number of letters correct will be recorded.

#### Eye tracking

A portable infra-red saccadometer (Ober consulting) will be fitted to the participant, which automatically presents a stimulus protocol and records the reaction times. Three sequences of stimuli (the step, antisaccade and Wheeless tasks) will be collected [71]. An additional substudy of participants (outside of the trial protocol), using an Eyelink 1000, will undertake the prosaccade and double-step tasks from the DEMoNS protocol [72]. These will be performed at screening visit 2 and week 28. These exploratory endpoints will be reported outside of the main trial manuscript.

### EDSS

All participants will have their disability evaluated by the EDSS, by a neurostatus-certified member of staff (doctor, specialist nurse or physiotherapist).

### Plans to promote participant retention and complete follow-up {18b}

As a research team, we will do our best to support participants’ retention in the trial. If treatment withdrawal of both IMPs occurs at any time point after randomisation, the participant will be asked if they would agree to continue with the trial assessments (both safety and efficacy). If withdrawal occurs within 2 months of starting IMP (whether or not the participant has taken any IMP during this time), the CI has the option of replacing that individual.

### Data management {19}

CCMR Two will use standardised procedures for data entry, coding, security, storage, and analysis to ensure data quality and confidentiality, as set out in the trial’s Data Management Plan v4.0. Source data will be recorded and subsequently entered electronically into MACRO, a secure and validated trial database which includes closed response entries and range restrictions to maximise valid data entry. This will be done contemporaneously, typically within 7 working days of completion of source documentation. Critical data points will undergo double data entry by different team members. Each participant will be assigned a unique trial ID to de-identify data. The trial coordinator and trial monitor will undertake quality control checks on data and issue queries in line with the trial-specific monitoring plan. Upon completion of the trial, the dataset will be locked, and analyses will be performed by the trial’s statistician. Regular audits of data quality and completeness will be conducted.

### Confidentiality {27}

All investigators and trial site staff involved in CCMR Two will comply with the requirements of the General Data Protection Regulation (GDPR) 2018, Data Protection Act 2018 and Trust policy with regards to the collection, storage, processing, transfer and disclosure of personal information and will uphold the act’s core principles. The trial staff will ensure that the participants’ anonymity is maintained. The participants will be identified only by initials, date of birth and a participant ID number on the case report form and any electronic database. All documents and data will be stored securely and only accessible by trial staff and authorised personnel. Access to this data will be restricted to authorised members of the trial team via a two-factor authentication system.

### Plans for collection, laboratory evaluation and storage of biological specimens for genetic or molecular analysis in this trial/future use {33}

Blood tests performed for this trial are as follows: full blood count (FBC), liver function tests (LFTs), urea and electrolytes (U&Es), C-reactive protein (CRP), and serum glucose. Vitamin B12 will be tested at the screening 2 visit. For female participants of childbearing potential, a serum pregnancy test will also be performed at the screening 2 visit. In addition to these tests, blood samples will be collected, processed and stored in − 80 °C freezers in the Clifford Allbutt Building for future analysis, if the participant has consented to this optional research sample collection. Samples will be labelled with trial-specific identifiers. Samples of blood, the components of blood (i.e. plasma/serum/cells), and stool samples may be shared with other collaborating academics or with industrial third parties (maybe both within and outside the UK and EEA where data protection laws are less strict) that are under contract to carry out sample analysis. Samples will only ever be used for research that is approved and deemed appropriate by an ethics committee. If there are unused samples left at the end of the trial, we will ask for ethical approval to use these in another project.

## Statistical methods

### Statistical methods for primary and secondary outcomes {20a}

A detailed statistical analysis plan (SAP version 1.0 (14 January 2025)) provides an account of the statistical methods for analysing the trial. Primary efficacy analyses will be based on the modified intent-to-treat (mITT) population with supplementary analyses with the PP population (definitions Sect. 20c).

### Primary outcome measure: full-field VEP

The primary analysis of this trial will compare the mean change in the P100 latency of the full-field VEP from baseline and week 26, between metformin + clemastine (research arm) and placebo (control arm). This will be based on eligible eyes with delayed (≥ 118 ms) baseline P100 latency. Eligible eyes will additionally have RNFL thickness ≥ 70 μm (see outcomes Sect. 12 for the definition of eligible eyes). A mixed-effect model will be used for eyes nested within participants, with random effects for participants and fixed effects for treatment allocation at randomisation, baseline P100 latency and stratification factors.

Fixed effects: treatment allocation, baseline P100 latency, age at consent (≤ 40 years vs > 40 years), sex (male vs female), baseline MS DMT treatment category (1 vs 2) [5], screening EDSS (≤ 4.0 vs > 4.0).

### Secondary efficacy analysis: multifocal VEP

The secondary (electrophysiology) outcome measure is the change in MF-VEP latency, between baseline and week 26, for those eyes with delayed (≥ 151 ms) latency at baseline. Eligible eyes will additionally have RNFL thickness ≥ 70 μm and ≥ 28/56 recordable segments (see outcomes Sect. 12 for the definition of eligible eyes). This will apply the same statistical analyses as for the primary outcome measure with the dependent variable replaced by the change in MF-VEP latency.

### Secondary efficacy analysis: change in lesional MTR

The secondary (MRI) outcome measure is the change in lesional MTR, between baseline and week 26, for the lesions stratified by location or by tissue-specific cohort baseline lesional MTR values.

Analyses will be presented for change in lesional MTR for MS lesions grouped according to each location as follows: periventricular, deep white matter, juxtacortical, leukocortical, cortical grey matter, deep grey matter, mixed deep grey matter and white matter, brainstem and cerebellar lesions. Based on previous research [27], we hypothesise that the clearest effect will be seen in grey matter and brainstem lesions. We will apply the same method as applied for the primary outcome measure, with the dependent variable replaced by the change in lesional MTR for each lesion location subgroup. An interaction term will compare treatment group differences between lesion locations.

Analyses of change in lesional MTR stratified by cohort baseline lesional MTR values will be applied using the same method as the primary outcome measure with the dependent variable replaced by the change in lesional MTR for lesions: (1) grouped as being above or below the cohort median lesion MTR and (2) grouped into quartiles based on the baseline cohort lesion MTR. In line with previous research [27], the interaction test between treatment effects in different lesional groups will be a variable of key interest.

### Interim analyses {21b}

The IDMC will review data, by treatment group, on the safety of participants in the trial. The IDMC will meet at least once a year, or at the frequency requested by the IDMC, until the end of the trial. There are no plans to stop the trial early for efficacy; therefore, there are no interim analyses for efficacy or futility. Efficacy data, by treatment group, will therefore not be routinely provided. The IDMC can request efficacy data, by treatment group, confidentially from the TSC if changes to the protocol are considered and these data are required by the IDMC to reach decisions about the ongoing risk/benefit to participants in the trial.

As described in the sample size section above, one pre-planned interim analysis was undertaken to review the sample size after the first 20 participants had completed their trial participation. It was through this mechanism that the sample size was increased from 50 to 70 participants (as approximately 50% of screened participants had only one eligible eye at screening).

### Methods for additional analyses (e.g. subgroup analyses) {20b}

The exploratory efficacy analyses will be performed at the level of the participant (mITT and PP populations) or eye (applicable to the primary outcome measure, unless otherwise specified in the SAP). Change in EDSS and neurofilament light will be analysed through linear regression. Analyses of changes in visual function and anatomy (VEP amplitude, visual acuity, colour vision, visual fields, optical coherence tomography) will use the same method as for the primary efficacy analysis.

## Methods in analysis to handle protocol non-adherence and any statistical methods to handle missing data {20c}

### Intent-to-treat (ITT) population

The ITT population is defined as all randomised participants regardless of whether they received randomised trial treatment.

#### Modified intent-to-treat (mITT) population

The mITT population is defined as all randomised participants in whom the replacement criteria were not met; that is, the duration of IMPs received being more than 2 months. The treatment group will be analysed as randomised at baseline. The unit for the primary efficacy analysis is per eye.

#### Per protocol (PP) population

The PP population is defined as those randomised participants who completed the 24 weeks of allocated treatment regardless of whether there were any dose or schedule modifications, except for participants who missed > 28 doses of either trial IMPs over the entire treatment period.

#### Safety population

The safety population comprises all participants who were randomised and received any dose of trial treatment.

#### Missing data

Missing data related to the primary outcome measure will be rare and will be accounted for using the mixed-effects models in the analyses. No imputation will be made for missing data.

#### Plans to give access to the full protocol, participant-level data and statistical code {31c}

Ownership of the data arising from this trial resides with the trial team. The CI will have full access to the final data set following completion of the analysis by the trial statistician. The protocol, statistical analysis plan and the deidentified dataset for the primary and secondary outcomes (with corresponding statistical codes) will be made available with publication from the Cambridge Clinical MS Research Group’s website (temporary web address at time of submission: https://www.cambridgeclinicalmsresearch.com/). Access requests should be made to the CI (Nick Cunniffe: ngc26@cam.ac.uk). A signed data access agreement will be required, and investigator support might be provided if part of an academic collaboration.

## Oversight and monitoring

### Composition of the coordinating centre and trial steering committee {5d}

The trial management group will run the trial day to day, and its members include CCMR Two investigators, trial coordinators, data managers, the trial statistician, and trial pharmacist. The TMG will meet monthly. Coordination and data management are performed by the neuroscience theme of the Cambridge Clinical Trials Unit.

Overall supervision of the trial will rest with the TSC. The TSC will consist of an independent Chairperson, independent physician experts, and an independent statistician, as well as representatives of the Sponsor and funder. The TSC will meet at least once a year and receive reports from TMG and IDMC. The charter details the operation of the TSC.

### Composition of the data monitoring committee, its role and reporting structure {21a}

The IDMC is independent of trial investigators, Sponsor and funder. The composition of the IDMC will be defined in the IDMC charter. The IDMC will meet at least every 12 months and will review the accumulated unblinded trial data for participant safety, trial conduct and progress, and it will make recommendations to the TSC concerning the continuation, modification, or termination of the trial. In addition, the IDMC will be asked to review the assumptions used in the sample size estimation when 20 patients have had their efficacy endpoints assessed, and advise whether a sample size re-estimation should be considered (see sample size, Sect. 14).

### Adverse event reporting and harms {22}

Recording of all AEs will start from the point of informed consent regardless of whether a participant has yet received a medicinal product. All AEs will be reported using MedDRA terminology version 24. Individual AEs will be evaluated by the investigator. This includes the evaluation of its seriousness and any relationship with the IMP(s). Expectedness will be assessed against the latest MHRA approved version of the reference safety information (RSI). Serious adverse events (SAEs) will be assessed by the CI in terms of expectedness and relatedness. SAEs are then notified to the Sponsor within 24 h of first notification. Suspected unexpected serious adverse reactions (SUSARs) will be subject to expedited reporting to the Sponsor, REC, and competent authority (MHRA), as detailed in the trial protocol.

### Frequency and plans for auditing trial conduct {23}

The investigator will make all trial documentation and related records available should an MHRA Inspection occur. Should a monitoring visit or audit be requested, the investigator will make the trial documentation and source data available to the Sponsor’s representative. All participant data will be handled and treated confidentially. The monitoring frequency is determined by an initial risk assessment prior to the start of the trial and a plan generated detailing the frequency and scope of monitoring for the trial.

### Plans for communicating important protocol amendments to relevant parties (e.g. trial participants, ethical committees) {25}

Protocol amendments will be reviewed and agreement received from the Sponsor for all proposed amendments prior to submission to the Health Research Authority (HRA), Research Ethics Committee (REC) and/or MHRA. The CI is responsible for communicating important protocol modifications to investigators, trial participants, registries and regulators. Before the start of the trial, we obtained ethical approval from the East Midlands—Nottingham 2 Research Ethics Committee (21/EM/0120).

### Dissemination plans {31a}

The results of the trial will be released either at oral presentation or trial publication, after a dissemination plan is agreed with the TSC. Summaries of results will also be made available to participants by means of a newsletter (communicated by email) and by means of an informal meeting with the trial team (virtual and in-person options available). Authorship decisions will be guided by uniform requirements for manuscripts submitted to medical journals (www.icmje.org).

## Discussion

While several clinical trials have deployed drugs capable of enhancing the differentiation of the OPC [23, 24, 26], the demonstration that metformin can reverse age-related changes in OPCs that prevent them from responding to such factors is an important development [30]. Consequently, there is a compelling rationale to test metformin, alongside pro-differentiation agents such as clemastine, in clinical trials of people with MS.

CCMR Two will test the hypothesis that metformin and clemastine can enhance remyelination in people with MS using both visual evoked potentials and magnetisation transfer ratio imaging; it is anticipated that in this way we will be able to detect the functional and structural consequences of remyelination with a sample size feasible for a single centre trial. We have applied our experience from the CCMR One trial—which showed that lesion remyelination in response to RXR agonism was dependent on its location and baseline tissue integrity[27]—and optimised our analyses to be sensitive to remyelination in those lesions that are more demyelinated at baseline and those that are located in the grey matter. We have additionally introduced selection criteria to yield a cohort that we expect is most likely to have a measurable between-group response in this trial setting.

There are, however, limitations to our chosen trial design. A particular challenge to translating promising preclinical research into remyelination trials is uncertainty about the optimum outcome measures to employ [73]. Our approach for CCMR Two has been to prioritise the VEP, given it has been directly confirmed to reflect myelin status in chronically demyelinated optic nerves [74] and has shown significant effects in three previous clinical trials [23, 24, 27], but to also include the MTR analyses that we think will be most sensitive to remyelination; in CCMR One, perhaps the most compelling evidence of a biological effect of bexarotene was the alignment between the imaging and electrophysiological results. We have also included several techniques that might be sensitive to, or represent the downstream consequences of, remyelination: MF-VEP [52], OCT [34], saccadometry [65], and LCLA [75]. Ultimately, all outcome measures in this field are exploratory, and several of this trial’s limitations stem from this point. In particular, in selecting only relapsing-remitting patients with visual pathway demyelination and focusing on remyelination of this particular tract, there follow uncertainties about the generalisability of results to the wider MS population. An additional limitation is the interpretation of a positive result. Would the primary effect be primarily attributable to the action of metformin, clemastine, or is there evidence of synergy between the two? There are pro-differentiation factors already present in demyelinated lesions [76] and metformin alone might be sufficient to achieve remyelination. Meanwhile, the modest effect on the VEP latency seen in the trial might indicate a mild pro-differentiation effect that can be better ‘unlocked’ by co-administration of metformin. CCMR Two is not designed to test these questions; the objective is to see if the remyelinating effect seen in animals translates into humans. Dissecting the different contributions of each drug and interrogating the possibility of synergy would require a much larger trial, demanding a multi-centre design, which would pose a particular challenge to using electrophysiological outcome measures [28, 73]. Questions would also remain as to how to deploy a remyelination treatment, such as whether it should be given continuously or periodically.

Despite this, CCMR Two represents an important step towards identifying a treatment capable of directly protecting neurons from degeneration, outside of the indirect effects of immunomodulatory disease-modifying treatments. As these drugs are widely used and known to be safe, this approach could then be readily deployed in confirmatory and phase 3 trials. If no treatment effects are shown, we are confident that the trial will have legacies that will lead to improvements in trial design and the development of optimum outcome measures.

## Trial status

Trial protocol 5.1, 27 March 2024. Statistical analysis plan 1.0, 14 January 2025. Recruitment began in March 2022 and was completed in October 2024. The trial is still ongoing at the time of submission, and analysis will begin in April 2025. The protocol paper was submitted for publication in March 2025 after recruitment closed, but this was to allow for the incorporation of the statistical analysis plan, which remained under revision until January 2025.

## Data Availability

The protocol, statistical analysis plan and the deidentified dataset for the primary and secondary outcomes (with corresponding statistical codes) will be made available with publication from the Cambridge Clinical MS Research Group’s website (temporary address at time of submission: https://www.cambridgeclinicalmsresearch.com/). Access requests should be made to the chief investigator (Nick G Cunniffe: ngc26@cam.ac.uk). A signed data access agreement will be required, and investigator support might be provided if part of an academic collaboration.

## Abbreviations

AE: Adverse event
AMP: Adenosine monophosphate
AMPK: AMP-activated protein kinase
ART: Automatic real time
ATP: Adenosine triphosphate
BBB: Blood-brain barrier
CCMR: Cambridge Centre for Myelin Repair
CI: Chief investigator
CONSORT: Consolidated Standards of Reporting Trials
CRF: Case report form
DIR: Double inversion recovery
DMT: Disease-modifying therapy/treatment
ECG: Electrocardiogram
EDSS: Expanded disability status scale
eGFR: Estimated glomerular filtration rate
GCP: Good clinical practice
GDPR: General Data Protection Regulation
GP: General practitioner
ICF: Informed consent form (ICF)
IDMC: Independent data monitoring committee
IMP: Investigational medicinal product
ISCEV: International Society for Clinical Electrophysiology of Vision
ITT: Intention to treat
LogMAR: Logarithm of the minimum angle of resolution
LCLA: Low-contrast letter acuity
LDL: Low-density lipoprotein
MAOI: Monoamine oxidase inhibitor
MedDRA: Medical Dictionary for Regulatory Activities
MF-VEP: Multifocal visual evoked potential
MHRA: Medicines and Healthcare products Regulatory Agency
MRI: Magnetic resonance imaging
MS: Multiple sclerosis
mTOR: Mammalian target of rapamycin
MTR: Magnetisation transfer ratio
NHS: National health service
OCT: Optical coherence tomography
OPC: Oligodendrocyte progenitor cells
PIS: Participant information sheet
PPI: Patient and public involvement
PP: Per protocol
RNA: Ribonucleic acid
RNFL: Retinal nerve fibre layer
RRMS: Relapsing-remitting multiple sclerosis
RXR: Retinoic acid X receptor
SAE: Serious adverse event
SAP: Statistical analysis plan
SD: Standard deviation
SR: Sustained release
SUSAR: Suspected unexpected serious adverse reactions
TSC: Trial steering committee
TMG: Trial management group
VEP: Visual evoked potential
